# A Bioreactor for Celullarised Membrane Culture and Delivery under Sterile Conditions

**DOI:** 10.3390/bioengineering11080785

**Published:** 2024-08-02

**Authors:** Ainitze Gereka, Uzuri Urtaza, Pablo Larreategi, Felipe Prosper, Enrique José Andreu, Ane Miren Zaldua

**Affiliations:** 1 Health Specialization, Leartiker S. Coop., 48270 Markina-Xemein, Spain; agereka@leartiker.com (A.G.); uurtaza@leartiker.com (U.U.); plarreategi@leartiker.com (P.L.); 2 Cell Therapy Area, Clinica Universidad de Navarra, 31008 Pamplona, Spain; fprosper@unav.es

**Keywords:** bioreactor, advanced therapies, cell culture, GMP manufacturing, operation room

## Abstract

A novel, user-friendly bioreactor for the cultivation of cellularised membranes for tissue engineering has been successfully designed, manufactured, and validated. This bioreactor features a culture vessel and a cover, the latter equipped with one or more sidewalls to ensure airtightness in two distinct zones, thereby maintaining sterile conditions. The cover, designed to integrate seamlessly with the culture vessel, includes several ports compatible with commercial connectors. This design allows the introduction of cells and culture medium without requiring the opening of the cover, thus preserving sterility. Additionally, the cover is equipped with flanges that effectively press the membrane against the bottom surface of the culture vessel, preventing it from shrinking or shifting. This ensures that cells can properly adhere to the membrane and proliferate. Manufactured under Good Manufacturing Practice (GMP) conditions, the bioreactor supports cultivation in optimal aseptic environments, thereby preventing external contamination. This feature is critical for the safe transportation of cultivated tissue to clinical settings. Validation tests have confirmed the bioreactor’s excellent performance, endorsing its suitability for intended applications in tissue engineering.

## 1. Introduction

In tissue engineering, the creation of an environment conducive to cell proliferation and tissue generation is paramount. This is typically achieved through the use of bioreactors, which maintain a controlled and sterile environment to facilitate cell attachment to a matrix within a sealed compartment [1]. Bilodeau et al. [2] conducted a comprehensive review analysing the advantages and disadvantages of various bioreactor designs tailored for specific tissue types. Their findings indicate that rotating-wall bioreactors are particularly suited for the culture of delicate tissues, whereas fixed-wall bioreactors are better suited for tissues with a complex geometry that are subjected to significant mechanical stresses in vivo. With respect to the matrix, polymeric materials are widely used as membranes in these bioreactors, providing surfaces that support cell attachment and growth, thereby mimicking the extracellular matrix to some degree [3,4,5,6,7,8]. Among the most prominent biodegradable polymers used for membrane production are Polycaprolactone (PCL) and Polylactic acid (PLA). A major challenge with these polymers is tailoring their properties to match the requirements of the target tissue. This issue can be addressed by incorporating hydrogels into the scaffold, enhancing its suitability for specific tissue engineering applications.

Even though submerging the scaffold in a media and adding hydrogels contributes to the material’s softening, the stiffness of these materials is generally higher than the target tissue [9]. This is why natural membranes are commonly used. Among them, collagen-based membranes (CBMs) have gained prominence in the market due to their biocompatibility and semi-permeability, which facilitate nutrient transfer [3]. A notable example of a CBM is the human amniotic membrane (hAM) derived from the human placenta. Thanks to its exceptional biocompatibility, decellularised hAM has found widespread application across various medical fields, including ophthalmology, dermatology, oral and maxillofacial surgery and otolaryngology [8]. Recent advancements in the stabilisation, preservation, and storage of tissues have led to the development of several commercially available hAM products. A comprehensive review by Gindraux et al. [10] provides detailed information on these products, underscoring the significant progress in this area. In recent years, the design of bioreactors used for tissue engineering has continued to progress in sophistication. The field of tissue engineering faces two primary challenges: (1) translating research-scale product designs into large-scale production of biologically-based products that are reproducible, safe, clinically effective, and economically acceptable and feasible [11,12]. This necessitates a combination of expertise in tissue biology and bioreactor design and manufacturing and (2) addressing regulatory issues for market placement. Given their nature, these implants could be classified as Class III devices by the FDA, functioning not only as traditional devices but also being biointeractive post-implantation [13].

In this context, the production of tissues that comply with Good Manufacturing Practices (GMPs) is essential. A critical aspect of a bioreactor design is minimising the number of aseptic operations, as each operation introduces potential contamination risks. Ideally, the entire tissue growth process, from cell expansion to the final product, should be accomplished in a single aseptic operation, including the transport to the surgical implantation location [14]. In the study carried out by Martin et al., they propose a methodology allowing hospitals and clinics to conduct autologous tissue engineering on-site, eliminating logistical issues associated with specimen transport [12]. Additionally, the development of novel, low-cost bioreactors could facilitate the widespread adoption of therapeutic approaches that might otherwise remain confined to research settings laboratories.

Achieving dynamic conditions that replicate both physiological and mechanical aspects of the body is essential for engineering most tissues [15]. Traditionally, preliminary experiments were conducted in Petri dishes before advancing to more complex dynamic bioreactors. In the regenerative medicine field, the cellularisation of collagen membranes serves as a therapeutic approach, emphasising the need to mimic physiological conditions, albeit not necessarily the mechanical needs [16]. Petri dishes offer advantages such as easy medium exchange and controlled gas conditions in an incubator, but they also present challenges, including potential contamination during transport from the laminar flow hood to the incubator and to the GMP lab or surgery room [17].

The objective of this study is to design, construct and try a bioreactor developed to achieve several critical goals: (1) replace Petri dishes in pivotal experiments, (2) scale up and translate research products to large-scale production, (3) minimise aseptic operations, and (4) culture various cell types on biocompatible membranes for biomedical research, somatic cell therapy and tissue engineering applications. The bioreactor is engineered to meet specific requirements, ensuring membrane secure fixation and providing a closed, aseptic environment conducive to sterile cell culture, thereby reducing the risk of contamination. Additionally, the bioreactor serves dual roles as both primary packaging for cell growth and final packaging for the transportation of the cellularised membranes to the surgical room, thus minimising the risk of healthcare-associated infections (HAIs) and the resulting cost savings to the healthcare system.

## 2. Materials and Methods

### 2.1. Materials

A medical and optical grade silicone has been selected for the manufacturing of this bioreactor, Lumisil^®^ LR 7601/60 A/B, from Wacker (Munich, Germany). It is a two-component Liquid Silicone Rubber whose technical properties are shown in Table 1; the properties have been measured on samples vulcanised for 15 min at 165 °C and cut from 2 mm thick plates.

Dulbecco’s Modified Eagle’s Medium (DMEM) was purchased from Sigma (Ref. D6429, Merck Life Science S.L., Madrid, Spain). This medium was used for CO_2_ exchange and sterility tests. Subsequently, for cell culture validation, a complete medium consisting of 10% Foetal Bovine Serum (Gibco, ref. 10101145, Life Technologies Europe BV, Bleiswijk, The Netherlands) and 50 µg/mL Gentamicin (GENTA-GOBENS-240, CIMA, Madrid, Spain) added to DMEM is used to check cell growth. Standard Cell culture flasks were purchased from Corning (Ref. 431080, Merck Life Science S.L., Madrid, Spain). The collagen membrane was a kind donation from Viscofan Bioengineering (Weinheim, Germany).

Adipose-derived human stem cells (ADSCs) were isolated as described by Arana et al. [21] from liposuction samples discarded from healthy donors after informed consent, fulfilling all serological criteria (negative test for Hepatitis B Virus; Hepatitis C Virus; HIV 1 and 2; Syphilis; HTLV I/II; HTLV I/II) and medical history to be an allogeneic donor (no oncological disease, no risk factors for infectious disease transmission, no prion diseases or diseases of unknown aetiology). ADSCs were cultured in a controlled incubator (HeraCell 240, Heraeus, Thermofixer Scientific SLU, Alcobendas, Madrid) at 37 °C in a 95% humidified atmosphere with 5.5% CO_2_. Further experiments were performed in a controlled incubator at 37 °C without a humidified atmosphere and without CO_2_ supply (B6 Function line, Heraeus).

**Figure 1:**
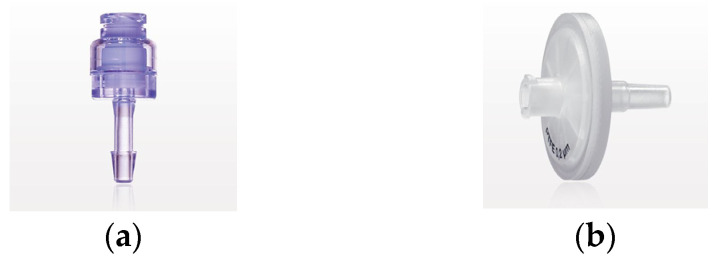
Photographs of the accessories: (**a**) Swabbable Female connector and (**b**) Female Luer Lock filter.

The bioreactor is designed in such a way that it can be fitted with any type of barb connectors and luer slip connectors if they have the required dimensions, such as 1/8-inch ID Tubing. Two main connectors were used for this study (Figure 1): (a) needless injection swabbable Female Luer Lock to Barb connector (Qosina, Ronkonkoma, NY, USA, Ref.: 80210) and (b) hydrophobic filter with Female Luer Lock Inlet and Male Luer Slip Outlet (Qosina, Ref.: 28246). Thanks to its swabbable valve, the first filter allows the entry of any liquid medium while maintaining the sterility of the bioreactors. The selected filters (Figure 1b) allow gas exchange to take place with the incubator chamber. This ensures optimum conditions for the cell culture and prevents medium evaporation, thus minimising possible medium losses. It is important to note that any connector chosen must ensure sterility to the innert part of the bioreactor to be a functional device, i.e., any connector with a cavity connecting the inside of the bioreactor to the outside is strongly discouraged.

### 2.2. Design

Viens et al. made a roadmap for the design of bioreactors in tissue engineering [18]. They proposed a four-step methodology: (1) identifying the needs and technical requirements, (2) defining and evaluating the related concepts, (3) designing the apparatus and drawing up the blueprints and (4) building and validating the apparatus.

#### 2.2.1. Design Requirements

As proposed by Viens et al. [18], the first step in the design methodology is to define the requirements. In particular, the bioreactors must provide the technological means with which to perform controlled studies; the design must allow (1) the holding of the membrane to prevent it from shrinking, (2) the nutrient supply, (3) the control of the physiological solution (pH, salt, and nutrient concentration), (4) the control of oxygen, (5) biocompatibility, (6) the maintenance of a sterile environment, and (7) the temperature control. In addition, it must be easy to use and handle and must comply with the GMP requirements.

The bioreactor design should be provided with an airtight and sterile chamber to grow different cell lines on biocompatible membranes and finally obtain a cellularised membrane to be used in various clinical conditions.

#### 2.2.2. Design of the Bioreactor

CREO Parametrics software (9.0.2.0) was used for the design of the devices, an application that includes collaborative solid modelling, assembly modelling, 2D orthographic views, finite element analysis, parametric modelling, and subdivision surface modelling, among others.

### 2.3. Device Fabrication

The device was injection moulded in a Liquid Silicone Rubber (LSR) machine, a horizontal and electrical 80 Tn machine from Engel, an e-victory 80 model, with a 20-litre dosing unit from ELMET, for dispensing both components of LSR, A and B. Mould temperature was fixed at 135 °C, whereas the injection barrel and the cold chamber were maintained at 18 °C. The vulcanisation time was 30 s. The injection was carried out in GMP conditions inside a classified and validated ISO 7 clean room according to ISO 14644-18 [22]. After that, the devices were post-cured at 200 °C in an oven for 4 h to avoid leaching problems. Before the validation test, the bioreactor was sterilised with Ethylene Oxide for 75 min at 54 °C, followed by aeration for 12 h.

### 2.4. Device Functionality Validation

To validate in vitro the functionality of the device, the following three different assays were proposed:

1. The device must ensure adequate watertightness. An internal experiment was conducted to ensure this characteristic;

2. The device must ensure an adequate gas exchange and preserve the cell culture sterility. These characteristics were validated at the same time in the same experiment. The test selected to validate the gas exchange was based on a characteristic of the culture medium that changes pH when the CO_2_ level is modified. The cell culture medium contains physiological pH buffers to maintain acidity around neutral pH. In addition, the culture media have an internal pH indicator called Phenol Red, which turns the colour of the media based on the pH: pink at pH 7.6, red at pH 7.4, orange at pH 7 and yellow at pH 6.5 [23]. The pH was measured with a Hanna pH meter, model HI 6221. The pH meter was calibrated before use with commercial standards pH 4, pH 7 and pH 10. The sterility tests were conducted in parallel at 48 and 96 h. The sterility test used was the automated detection by the Bact/Alert^®^ system by BioMerieux (Biomerieux España, SA, Madrid, Spain) following the procedures described by the European Pharmacopoeia (monograph 2.6.27) [24]. The sterility test was performed with an aliquot of the culture medium used in the experiments;

3. The device must ensure a homogeneous cell culture, allowing a correct assembly and fixation of the biocompatible membranes.

## 3. Results

### 3.1. Bioreactor Design

**Figure 2:**
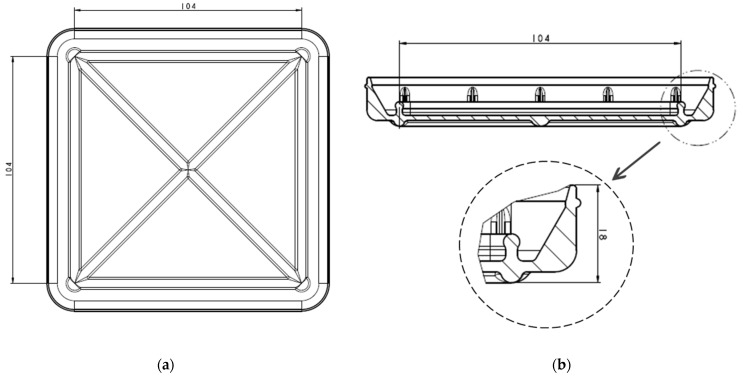
Vessel design with dimensions in mm: (**a**) Bottom view of the vessel; (**b**) section view and enlarged sealing zone.

A bioreactor with a flexible behaviour for cellularised membrane cultivation was designed and patented (EP3719111B1 [25]). Bioreactors of two sizes have been developed. This paper presents the design and size of the larger bioreactor, which has a volume of about 92 mL. The smaller one has been developed for research purposes and has a volume of 4 mL. It consists of a vessel (Figure 2a) with a surrounding chamber with specially designed sidewalls to ensure watertightness and ribs at the bottom to give rigidity to the device. In Figure 2b, the section of the vessel can be seen, with two sealing zones enlarged for more details on the design.

**Figure 3:**
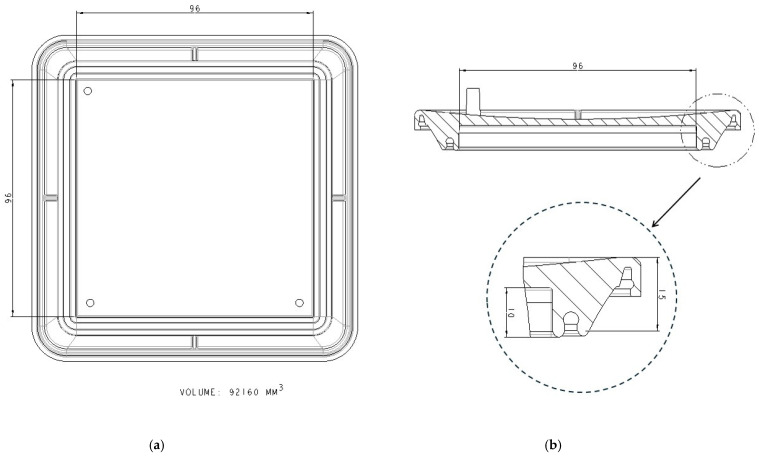
Cover design with dimensions in mm: (**a**) Bottom view of the cover; (**b**) section view and enlarged sealing zone.

**Figure 4:**
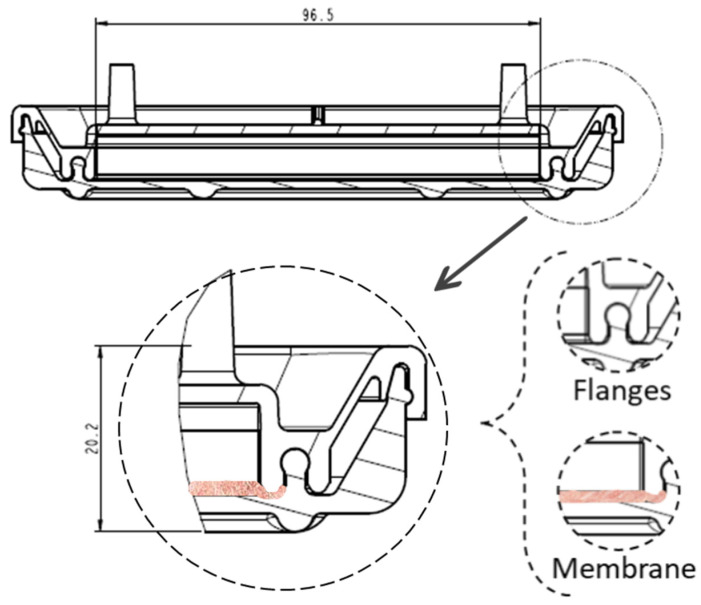
Membrane location once the cover and the vessel are adjusted, with dimensions in mm.

The device has a cover with two sidewalls with several flanges specially designed for fitting in the sealing zones of the vessel (Figure 3) and three ports on which the commercial connectors and filters will be inserted. In Figure 3b, the section of the cover can be seen, with the two sealing zones enlarged. The internal flange of the inside sealing zone (Figure 3b) is a little bit longer than the external flange to allow the membrane to be kept in position. Additionally, as the cover and vessel are fitted, the inner flange (Figure 4) may press the cell culture membrane previously located over the bottom inner surface of the vessel. Undesired displacements of the membrane are thus avoided. It is noted that the pressing of the cell culture membrane against the bottom surface of the culture vessel is performed using an element which already forms part of the locking mechanism to attach the cover to the culture vessel. Further elements to keep the membrane in position are thus avoided. It should be observed that a suitable culture space is formed within the device once the cover and the vessel are attached to each other. Particularly, the culture space may be provided such that, in use, a culture medium may cover the membrane and, at the same time, at least a thin layer of air may be located between the base of the cover and the culture medium covering the membrane, to avoid the culture medium adhering to the cover.

Once the cover and the vessel are attached to each other, providing a double sealing against external pollution, cells could be cultivated over the membrane in an aseptic environment. The three access ports can be used to introduce the cells and the liquid media for the cultivation, and the filter will allow the gas exchange with the surrounding environment.

### 3.2. Bioreactor Validation

#### 3.2.1. Watertightness

**Figure 5:**
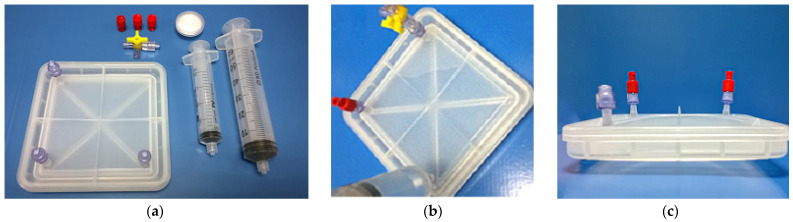
Watertightness trial: (**a**) Closed bioreactor and accessories; (**b**) filling water in the closed bioreactor with 2.6 times its volume; and (**c**) cross view of the filled bioreactor.

To ensure an airtight environment, a simple leakage test was carried out. After the closure of the bioreactor, a water content of 2.6 times the volume of the bioreactor was introduced in it, applying a pressure of 250 Pa (Figure 5a,b). The idea was to evaluate the pressure that the device can withstand without suffering leakage from the cavity to the surrounding area. In this test, the water did not cross the first sealing zone, so it can be concluded that the device is an effectively sealed system (Figure 5c). The bioreactor can withstand an internal pressure of two and a half times its volume without losing watertightness.

#### 3.2.2. Gas Exchange and Device Sterility

**Figure 6:**
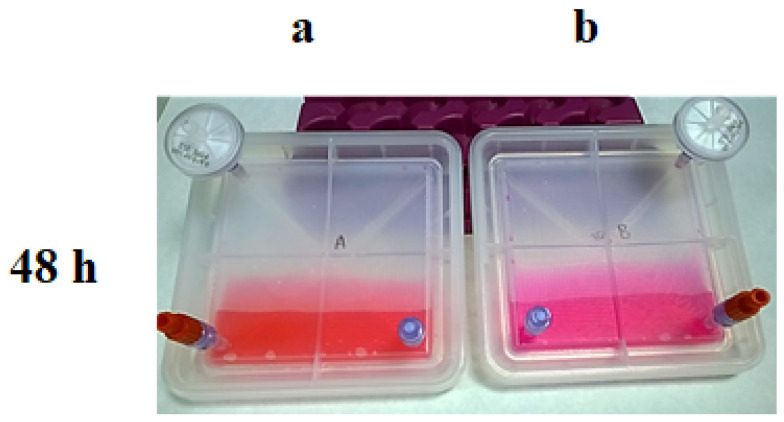
Results of the CO_2_ exchange experiment at 48 h; Culture media colour in each bioreactor after being kept in an incubator: (**a**) with 5% of CO_2_; (**b**) without CO_2_.

The test selected to validate the gas exchange was based on the properties of the culture medium to change the pH due to levels of CO_2_. The CO_2_, in the gas phase, dissolves in the medium, establishing a balance with the hydrogen carbonate ions and stabilising the pH. In this validation, the device is tested with culture medium without antibiotics or serum because the antibiotics can inhibit growth and mask the sterility test. In addition, serum can act as a natural buffer medium, and the pH variation could be masked. To ensure the proper exchange of CO_2_ in the device, the following experiment was proposed (Figure 6):

Step 1: Fill two devices (final prototype) with 30 mL of DMEM media, which is a high glucose medium, with carbonate–bicarbonate buffer at pH 7.2–7.4 and Phenol Red as an indicator. No antibiotics or foetal bovine serum (FBS) were added, considering that antibiotics would interfere with the sterility and FBS would affect the pH;

Step 2: Device A was maintained at 37 °C with 5% of CO_2,_ whereas device B was kept at 37 °C in an incubator without CO_2_ input. Then, 48 h later, samples of both conditions were extracted and analysed to check the pH and the sterility. Media from device A maintained the expected pH; however, the lack of CO_2_ input in device B turned the pH into an alkaline pH (Figure 6).

**Figure 7:**
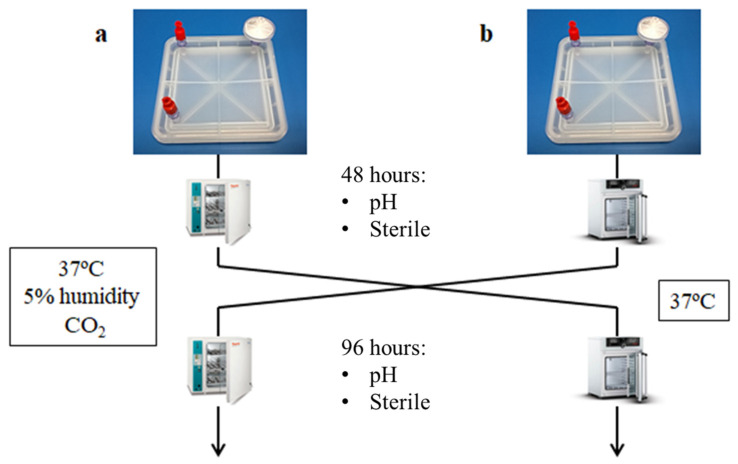
Scheme of the CO_2_ exchange experiment: (**a**) Devices are kept 48 h in an incubator with 5 % CO_2_ and then they are exchanged to an incubator without CO_2_ for 48 h more; (**b**) Devices are kept 48 h in an incubator without CO_2_ and then they are exchanged to an incubator with 5 % of CO_2_ for 48 h more.

**Figure 8:**
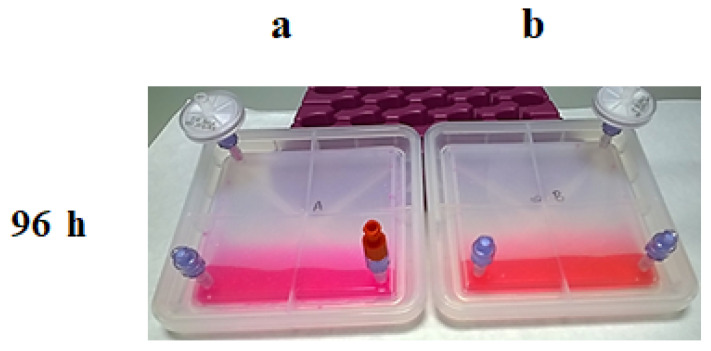
Results of the CO_2_ exchange experiment at 96 h; Culture media colour in each bioreactor after being kept in an incubator: (**a**) without CO_2_; (**b**) with 5% of CO_2_.

Step 3: Then, the positions were exchanged (Figure 7), and after an additional 48 h, the same analysis was performed. Device B, at 37 °C and with 5% of CO_2_, recovered the normal pH; in contrast, the media in device A, placed at 37 °C without CO_2_ input, turned basic (Figure 8). 

Two devices were used in each condition (A1, A2, B1, B2), and the average of three measurements of pH in both bioreactors was calculated. The average and standard deviation of pH due to steps 2 and 3 are shown in Table 2.

The sterility tests were conducted in parallel for 48 and 96 h. The results of the microbiological examination were made following the European Pharmacopoeia recommendations [24] and were negative in all cases (Table 3).

In this simple assay, it has been demonstrated that the device is able to exchange CO_2_ efficiently and that the sterility of the culture is maintained.

#### 3.2.3. Cell Culture Validation

Finally, an in vitro test was designed to demonstrate that the device can carry out the processes under specific conditions according to its intended uses. The study proposed was designed to demonstrate that the device fulfils its function, the formation of cellularised membranes in a comparable way to the growth obtained with standard 175 cm^2^ culture flasks. The in vitro proof of concept study was performed with a focus on a specific type of membrane (collagen) and cell line (hMSC), which is described below.

The cells were obtained from healthy volunteers who had signed the informed consent for the donation, and the expansion was performed following the normalised working procedures established in the advanced therapy laboratory of the Clínica Universidad de Navarra.

The bioreactor, composed of medical-grade silicone, was assembled, and the collagen membrane (100 cm^2^), previously equilibrated with the culture media, was fixed. Cells were plated at a concentration of 5000 cells/cm^2^, resulting in a total of 500,000 cells in a final volume of 30 mL of culture media. The same concentration of cells was used with the standard culture flask, but since the flask has a bigger surface area, the total number of cells was higher: 875,000 cells in a final volume of 30 mL of culture media. In this case, a complete culture media is used with antibiotics and serum (DMEM + 10% FBS + 50 μg/mL Gentamicin).

Both cultures were kept in an incubator under the same conditions: 37 °C, 5% CO_2_ and a humid atmosphere. On day 3, the culture media were changed. On day 7, cells from both cultures were harvested, and cells were manually counted in a Neubauer chamber. Cell viability was checked using Trypan Blue. Table 4 shows the summary of the results obtained in the proliferation experiment.

The viability achieved with the device was high (more than 95%) and comparable to the standard flask culture method. Apart from that, the highest performance, considering the number of cells per cm^2^, was obtained with the device designed in this work.

## 4. Discussion

An easy-to-use and easy-to-handle bioreactor has been designed, manufactured, and validated for cell culture on a membrane and its transport to the surgical room under sterile conditions. The design of the bioreactor and its manufacture using flexible and elastic materials, such as silicone, allows the membrane to be fixed inside the vessel, preventing its shrinkage when the culture medium is introduced into the device. The bioreactor is not recyclable because it is a crosslinked silicone, but it can be reused after cleaning and sterilisation (silicone resists all types of sterilisation methods).

A simple test to demonstrate the tightness of the device has been designed and verified. If a volume of water 2.6 times the volume of the cavity is introduced, the water does not pass through the first sealing zone of the device to the second sealing zone, indicating that the design ensures good sealing. However, the device shall not be completely filled with medium, as it is recommended to have a thin layer of air between the membrane and the base of the cover to ensure good cell culture conditions.

The gas concentration and temperature are controlled by introducing the bioreactor into an incubator chamber. The filter inserted in one of the ports in the cover allows gas exchange. The colour change of the medium and pH measurements confirmed that the gas exchange was performed correctly. In addition, the microbiological examinations of aliquots of the medium according to the recommendations of European Pharmacopoeia [24] were negative, indicating that the sterility of the culture was preserved.

The in vitro proof of concept study carried out on a specific type of membrane (collagen) and cell line (hMSC) concluded that the viability achieved with the device was high (more than 95%) and comparable to the standard flask culture method. However, the newly developed bioreactor has the following advantages over culture flasks: (1) Minimises the number of operations that may have a risk of losing the aseptic conditions. With the commercial valves that can be attached to the ports in the cover, users do not have to open the bioreactor when they want to change the medium; (2) avoids shrinkage problems: when working with collagen membranes, they usually shrink when the medium is introduced. The cover design allows the membrane to be held in place; (3) logistics: The bioreactor could be used to transport the sample to the surgical room. The bioreactor allows the transport in aseptic conditions.

## 5. Patents

A European patent was applied, “Devices for cellularized membrane cultivation and kits”; EP3719111B1 [25].

## Figures and Tables

**Table 1:** Technical data of Lumisil^®^ LR 7601/60.

Property	Value	Method
Hardness Shore A	60	DIN ISO 48-4 [18]
Tear strength	15 N/mm	ASTM D 624 B [19]
Tensile strength	7.3 N/mm^2^	ISO 37 type 1 [20]
Elongation at break	290%	ISO 37 type 1 [20]

**Table 2:** The average and standard deviation of pH test results (n = 3 in each bioreactor).

	A Series	B Series
48 h	7.54 ± 0.04	8.76 ± 0.01
96 h	8.73 ± 0.08	7.46 ± 0.01

**Table 3:** Sterility test results.

Property	Device A	Device B
48 h	Correct	Correct
96 h	Correct	Correct

**Table 4:** Cell culture results.

	Total n° Cells	Performance of Cell Culture (n° Cells/cm^2^)	% Viability
Bioreactor under development (100 cm^2^)	7.3 × 10^6^	73,000 cells/cm^2^	>95
Standard culture flask (175 cm^3^)	9 × 10^6^	51,500 cells/cm^2^	>95

## Data Availability

The original contributions presented in the study are included in the article; further inquiries can be directed to the corresponding author/s.
